# Anthelmintic activity of *Carica pubescens* aqueous seed extract and its effects on rumen fermentation and methane reduction in Indonesian thin-tailed sheep: An *in vitro* study

**DOI:** 10.14202/vetworld.2023.1421-1428

**Published:** 2023-07-09

**Authors:** Zein Ahmad Baihaqi, Irkham Widiyono, Amado A. Angeles, Bambang Suwignyo, Wisnu Nurcahyo

**Affiliations:** 1 Post Doctoral Program, Department of Internal Medicine, Faculty of Veterinary Medicine, Universitas Gadjah Mada, Yogyakarta, Indonesia; 2 Research Center for Animal Husbandry, National Research and Innovation Agency (BRIN), Indonesia; 3 Department of Internal Medicine, Faculty of Veterinary Medicine, Universitas Gadjah Mada, Yogyakarta, Indonesia; 4 Institute of Animal Science, College of Agriculture and Food Science, University of the Philippines Los Baños, Philippines; 5 Department of Animal Nutrition, Faculty of Animal Science, Universitas Gadjah Mada, Yogyakarta, Indonesia; 6 Department of Parasitology, Faculty of Veterinary Medicine, Universitas Gadjah Mada, Yogyakarta, Indonesia

**Keywords:** *Carica pubescens*, fermentation, greenhouse, *Haemonchus contortus*, metabolite, ruminant

## Abstract

**Background and Aim:**

Seeds from *Carica pubescens* were observed to be abundant as waste. This waste contains active plant compounds whose utilization has not been studied in the livestock sector. This study was conducted to evaluate the potential of an aqueous seed extract of *Carica pubescens* (ASE) as an anthelmintic agent during rumen fermentation and methane reduction.

**Materials and Methods:**

Aqueous seed extract of *Carica* *pubescens* was prepared from *C. pubescens* cultivated in Wonosobo, Indonesia. Phytochemical analyses were performed to quantify the secondary metabolite content of ASE. *In vitro* adult worm mortality tests, scanning electron microscopy, and gas production tests were conducted to evaluate rumen characteristics, methane reduction, and the potential of ASE as an anthelmintic against *Haemonchus contortus*. Adult worms and ruminal fluid were collected from Indonesian thin-tailed sheep. Two-way analysis of variance followed by Tukey’s test was performed using the Statistical Package for the Social Sciences^®^ 21.0 software to detect significant differences.

**Results:**

*In vitro* study results showed that 1–5% ASE inhibited *H. contortus* after the 1^st^ h of incubation, and 5% ASE suppressed 100% of adult *H. contortus* worms in the 5^th^ h. Scanning electron microscopy analysis of ASE-treated worms ASE revealed damaged cuticle structures. ASE had no significant effect on pH, NH_3_, volatile fatty acid, acetate, propionate, butyrate, acetate: propionate, or microbial protein in rumen fluid (p *>* 0.05). The *in vitro* feed fermentation results showed that ASE significantly affected methane reduction.

**Conclusion:**

The inclusion of up to 5% ASE in sheep diets may serve as a potential alternative anthelmintic against *H. contortus* as well as a methane reduction agent, without deleterious effects on rumen fermentation.

## Introduction

Parasitic nematodes are important pathogens that infect animals and cause significant economic losses worldwide. *Haemonchus contortus* (barber pole worm) is one of the most pathogenic nematodes of ruminants [1]. Gastrointestinal nematodes (GINs) are important pathogens of small ruminants that cause significant losses to these livestock species [2]. Sepúlveda-Vázquez *et al*. [3] added that commercial anthelmintics is a treatment that is widely used in parasite control, but in the tropics, GIN populations are resistant to one or more classes of these drugs. Gastrointestinal nematode infection is a major health problem in small grazing ruminants. *Haemonchus contortus* is known to occur in Indonesia, and its high prevalence has been recorded in the Wonosobo Regency during the dry and rainy seasons. The tropical environmental conditions of Indonesia constitute an ideal habitat for parasitic species [4].

Livestock plays a crucial role in food and nutritional security. However, livestock production accounts for 18% of global greenhouse gas (GHG) emissions [5]. Cheng *et al*. [6] stated that climate is changing globally, affecting livestock production. Climate affects livestock growth rate, milk and egg production, reproductive performance, morbidity, mortality, and feed supply. Simultaneously, livestock is a climate change driver, generating 14.5% of total anthropogenic GHG emissions. Mazinani *et al*. [7] reported that most dietary proteins in the rumen are rapidly degraded into ammonia. Ammonia can be absorbed through the rumen wall, which increases the blood urea levels. The remainder of the urea or ammonia that is not used by livestock will be excreted in the urine. One strategy for protecting feed proteins is protein bypass, which increases the amount of protein entering the abomasum.

Legumes are a high-quality feed with high crude protein (CP) content [8]. Moreover, legumes are a source of tannins, which are a source of amino acids for ruminants and non-ruminants [9], and may have a positive effect on gastrointestinal morphology and production [10, 11]. Almost all legumes contain active plant compounds such as in *Paraserianthes falcataria* stem bark waste, which contains tannins, flavonoids, alkaloids, saponins, and steroids that have the potential to inactivate *H. contortus*. This was evidenced by the results of mortality tests, structural changes, and damage to the cuticle and longitudinal reticular back of the worm [5]. Moreover, Baihaqi *et al*. [12] stated that active plant compounds, such as condensed tannin (CT) and hydrolyzable tannin (HT), reduced methane production. Both play an important role in reducing methane; however, CT is more widely studied due to its high prevalence. The concept of using tannins still presents its own challenges in terms of the dose administered, the structure of the tannin itself, the substitution of other ingredients, and the type of livestock treated. Niderkorn and Jayanegara [13] reported that plant bioactive compounds increase nitrogen-use efficiency by increasing rumen-undegradable proteins, which are often called rumen bypass proteins, thereby providing protection from excessive microbial protein degradation in the rumen. Due to the presence of tannins, feed with a high CP content, such as legume alfalfa [8, 9], can be digested in the abomasum rather than in the rumen [14]. Ebeid *et al*. [15] and Suwignyo *et al*. [8] reported that researchers have focused on using plant bioactive compounds as alternative feed additives to alter rumen characteristics, increase protein metabolism, and decrease CH_4_ production. Chai *et al*. [16] showed that rambutan seeds contain active saponins and tannins that are discarded during fruit processing. The *C. pubescens* fruit processing industry in Indonesia is mostly managed by small- and medium-sized enterprises. Most of the final disposal of fruit industry waste in Indonesia is not managed, causing environmental pollution. One of the most abundant types of waste is the seeds of this plant. Rahayu *et al*. [17] demonstrated that *C. pubescens* fruit contains active compounds such as flavonoids, alkaloids, and phenolic compounds. However, the active compounds in *C. pubescens* seeds have not been studied, even though they are abundant.

Therefore, in this study, we investigated the potential of *C. pubescens* seed extract as an alternative anthelmintic, as well as its effects on rumen microbial fermentation and methane reduction in small ruminants.

## Materials and Methods

### Ethical approval

The study was approved by the Institutional Ethical Committee of the Faculty of Veterinary Medicine at the Universitas Gadjah Mada, Yogyakarta, Indonesia. Number: 0013/EC-FKH/Int./2019.

### Study period and location

The study was conducted from November 15, 2022, to January 31, 2023. The *in vitro* anthelmintic studies were performed at the Animal Parasitology Laboratory in the Department of Internal Medicine, Faculty of Veterinary Medicine, Universitas Gadjah Mada, while *in vitro* rumen fermentation studies were carried out at the Nutritional Biochemistry Laboratory, in the Department of Animal Nutrition and Feed Science, Faculty of Animal Husbandry, Universitas Gadjah Mada.

### Plant material and extraction

*Carica pubescens* (herbarium voucher number: PPN-cc-003) seeds were collected from small- and medium-sized enterprises (CV. Gemilang Kencana,Wonosobo, Central Java, Indonesia). For the aqueous extracts, 150 g of powdered seed material was macerated with 750 mL of distilled water and incubated for 24 h. Macerated extracts were then filtered on cotton and concentrated under reduced pressure in a rotary evaporator at 40°C–50°C before being stored at 4°C. The dry extracts were subjected to phytochemical screening to identify the major phytochemical groups [18].

### Determination of plant phytochemicals

Qualitative detection was performed on *C. pubescens* seeds to detect active compounds such as tannins, alkaloids, flavonoids, and steroids using the procedure described by Trease and Evans [19]. Seed identification was performed under the direction of Professor Suwijiyo Pramono of Pharmaceutical Biology, Universitas Gadjah Mada. The total phenolic content in the plant extracts was measured using the Folin-Ciocalteu method, and the results were expressed as mg gallic acid equivalents [20]. The total flavonoid content of plant waste extracts was determined using a colorimetric method, and the results were expressed as milligram rutin, as previously reported by Nabavi *et al*. [21].

### *In vitro* adult worm mortality test

Adult female *H. contortus* worms were collected from the Godean sheep abattoir, Yogyakarta. *In vitro* anthelmintic studies were conducted using a combination of methods [22]. *Haemonchus contortus* worms were divided into four treatment groups (aqueous seed extract of *Carica* *pubescens* [ASE] 0%, ASE 1%, ASE 2.5%, and ASE 5%) and a positive control group with 0.2% albendazole (20 worms per group). The worms were placed in a Petri dish (50 mm) with extract or albendazole solution, and incubated at 37°C for 0.5, 1, 2, 3, 4, 5, and 6 h. The procedure was conducted 3 times and repeated on different days. The incubation time at which worms died was recorded. The number of surviving worms was recorded and their death was ensured by pressing the worm’s body with a tweezer.

#### Scanning electron microscopy

*Haemonchus contortus* obtained from the *in vitro* assay studies were fixed with 2% glutaraldehyde solution in a 0.1 M sodium cacodylate buffer for 4 h at 4°C. The worms were dehydrated with ethanol (30% – absolute), critical-point dried with an EMSCOPE CPD 750 (Ashford, Great Britain), and coated with gold-palladium for 5 min. Parasites were observed using an S450 scanning electron microscope (Hitachi, Tokyo, Japan) at an accelerating voltage of 15 kV.

### *In vitro* gas production test

In this study, the fermented substrate consisted of 60% elephant grass (*Pennisetum purpureum* cv Mott), 40% concentrate, 30% wheat pollard, and 10% soybean meal. The chemical compositions of the grass and concentrate are shown in Table-1. Rumen fluid was obtained from the rumen of Indonesian thin-tailed sheep in a Godean slaughterhouse (Mutiara Domba, Sleman, Indonesia). Sheep were previously fed for 1 month with *P. purpureum*, a brand of wheat, and soybean meal, based on 64% total digestible nutrient and 16.6% CP.

**Table-1:** Chemical composition of *P. purpureum*, soybean meal, and wheat pollard.

S. No.	Feed ingredients	Dry matter	Ash	CP	Crude fat	Crude fiber
1	*P. purpureum*	23.7	13.4	9.72	4.22	38.9
2	Soybean meal	89.3	9.24	45.3	3.3	6.2
3	Wheat pollard	87.8	4.23	17.9	4.21	8.71

CP=Crude protein, *P. purpureum=Pennisetum purpureum*

The treatment diets were P0 (300 mg fermented substrate), P1 (300 mg fermented substrate + 1% ASE), P2 (300 mg fermented substrate + 2.5% ASE), and P3 (300 mg fermented substrate + 5% ASE). The mixtures of feed samples and ASE, according to the treatment groups were incubated in a syringe containing buffered rumen fluid in triplicate, as described by Menke and Steingass [23]. Calibrated 100 mL glass syringes containing approximately 300 mg of sample were prewarmed at 39ºC before being infused with CO_2_ gas and 30 mL of buffered rumen fluid. The mixtures were incubated in a water bath at 39°C for 48 h. After incubation, gas production was measured before (0 h) and at 2, 4, 6, 8, 12, 24, 36, and 48 h. The fermentation kinetics were estimated using a fit curve program, as described by Hariadi and Santoso [24]. The ruminal pH was measured using a pH meter, and rumen fluid samples for rumen fermentation parameters were collected at the end of the incubation period. The concentration of NH_3_ was determined using the method described by Solorzano [25], and volatile fatty acid (VFA) was measured using gas chromatography according to the Association of Official Analytical Chemists [26]. Microbial proteins were quantified using the method described by Yulis *et al*. [27]. Methane was determined by administering NaOH at the end of the incubation period, as described by Yusuf *et al*. [28].

### Statistical analysis

Data from the worm mortality test, fermentation, and gas production parameters were analyzed using a one-way analysis of variance. Statistical differences between the means were determined using Duncan’s multiple-range test. Results were considered statistically significant at p < 0.05. The analyses were performed using IBM Statistical Package for the Social Sciences version 20 (IBM Corp., Chicago, USA). Data are presented as mean ± standard deviation (SD).

## Results

The qualitative phytochemical analyses of the ASE are presented in Table-2. The results of the quantitative analyses of total phenols, flavonoids, and tannins in the ASE are presented in Table-3. The observed total phenolic content was 9.5 mg gallic acid equivalent/g DW, whereas the total flavonoids, tannins, CT, and HT contents were 3.1 mg RE/g DW, 5.4%, 3.8%, and 2.9%, respectively.

**Table-2:** Qualitative phytochemical analyses of ASE of *C. pubescens*.

Secondary metabolite	ASE of *C. pubescens*
Tannin	+
Flavonoid	+
Alkaloid	+
Saponin	+
Steroid	+

*C. pubescens*=*Carica pubescens*, ASE=Aqueous extract of seed extract of *C. pubescens*

**Table-3:** Total phenol, flavonoid, and tannin content of ASE of *C. pubescens*.

Material	Flavonoids content (mg RE/g dw)	Total phenolic (mg GAE/g dw)	Tannin total (%)	CT (%)	HT (%)
*C. pubescens* ASE	3.1	9.5	5.4	3.8	2.9

*C. pubescens*=*Carica pubescens*, GAE=Gallic acid equivalent, CT=Condensed tannin, HT=Hydrolyzed tannin, ASE=Aqueous seed extract of *C. pubescens*

**Figure-1:**
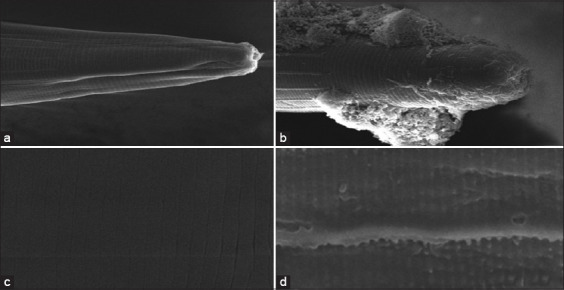
Scanning electron microscopy images of *Haemonchus contortus*. (a and c) Control *H. contortus* worms were smooth, free of aggregates on the cuticle, and had an undamaged reticular longitudinal back. (b) After treatment with aqueous seed extract of *Carica* *pubescens* (ASE), *H. contortus* had a damaged buccal area (buccal cavity). (d) *H. contortus* body had wrinkles in the annular cuticles after treatment with ASE.

Table-4 shows that ASE at a concentration of 1–5%, ASE inhibited *H. contortus* in the 1^st^ h. At a concentration of 5% (50 mg/mL), ASE successfully killed 100% of the worms (p = 0.05) at the 5^th^ h, whereas albendazole 2 mg/mL killed 100% *H. contortus* at the 2^nd^ h. The suppression of *H. contortus* nematodes by ASE at various concentrations showed that the 100% mortality rate of *H. contortus* at a concentration of 50 mg/mL at the 5^th^ h cannot be separated from the presence of active compounds in the seeds of the plant. Scanning electron microscopy images of the anterior end and cuticle of adult female *H. contortus* are presented in Figure-1.

**Table-4:** *In vitro* *H. contortus* mortality test with ASE of *C. pubescens*.

Treatment	The percentage of worm mortality at the incubation time						

	0.5 h	1 h	2 h	3 h	4 h	5 h	6 h
ASE 0%	0 ± 0.00	0 ± 0.00	0 ± 0.00	0 ± 0.00	0 ± 0.00	0 ± 0.00	0 ± 0.00
ASE. 1%	0 ± 0.00	3 ± 0.57	13 ± 1.15	33 ± 0.57	53 ± 0.57	73 ± 1.52	83 ± 1.52
ASE 2.5%	03 ± 0.57	26 ± 1.52	43 ± 1.52	56 ± 1.15	76 ± 0.57	83 ± 0.57	86 ± 1.15
ASE 5%	13 ± 1.15	23 ± 1.15	36 ± 1.15	56 ± 1.15	86 ± 1.52	100 ± 0.00	100 ± 0.00
Albendazole 0.2 %	4 ± 1.00	8 ± 2.00	100 ± 0.00	100 ± 0.00	100 ± 0.00	100 ± 0.00	100 ± 0.00

ASE=Aqueous seed extract of *C. pubescens*, *H. contortus=Haemonchus contortus, C. pubescens=Carica pubescens*

The results of the *in vitro* feed fermentation study are shown in Table-5. The ASE had no significant effect on the pH, NH_3_, total VFA, acetate, propionate, butyrate, acetate: propionate ratio, or microbial proteins in the rumen fluid (p *>* 0.05). As shown in Table-5, there was no significant effect of ASE treatment up to 5% on the microbial protein concentration (p > 0.05).

**Table-5:** Effects of the addition of ASE on rumen pH, NH3, VFA, and microbial protein parameter (*in vitro*).

Rumen fermentation parameters	Treatment group			

	P0 (ASE 0%)	P1 (ASE 1%)	P2 (ASE 2.5%)	P3 (ASE 5%)
pH^ns^	6.86 ± 0.05	6.82 ± 0.48	6.84 ± 0.51	6.81 ± 0.08
NH3 (mg/100 mL)^ns^	28.38 ± 0.02	28.26 ± 0.65	28.08 ± 0.14	27.94 ± 0.03
Total VFA (mM)^ns^	104.32 ± 1.91	103.86 ± 2.04	102.38 ± 1.62	101.91 ± 2.34
Acetate (%)^ns^	78.63 ± 0.82	78.41 ± 0.94	78.11 ± 0.47	77.96 ± 0.31
Propionate (%)^ns^	20.38 ± 0.94	19.71 ± 0.42	19.37 ± 0.46	19.08 ± 0.81
Butyrate (%)^ns^	13.52 ± 0.17	13.47 ± 0.21	13.38 ± 0.37	13.84 ± 0.32
Acetate:Propionate^ns^	3.81 ± 0.13	3.97 ± 0.17	3.98 ± 0.12	3.76 ± 0.14
Microbial protein (mg/mL)^ns^	0.79 ± 0.02	0.76 ± 0.01	0.75 ± 0.05	0.72 ± 0.01

P0=300 mg fermented substrate, P1=300 mg fermented substrate + 1% ASE, P2=300 mg fermented substrate + 2.5% ASE, P3=300 mg fermented substrate + 5% ASE, ASE=Aqueous seed extract of *Carica* *pubescens*, ns=Non significant (p > 0.05), VFA=Volatile fatty acids

**Figure-2:**
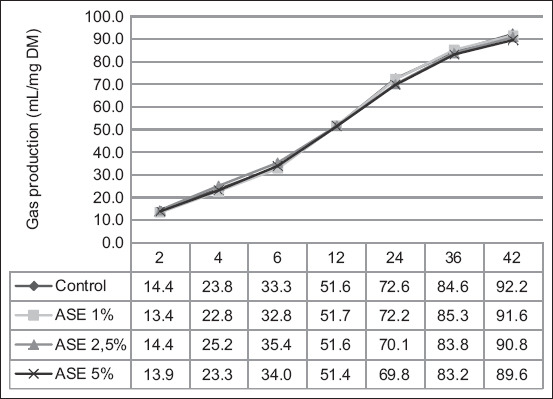
Effects of aqueous seed extract of *Caric*a *pubescen*s (0%, 1%, 2.5%, and 5%) in diet on gas production profiles during *in vitro* rumen fermentation.

The effects of ASE addition on rumen gas production kinetics are shown in Figure-2 and Table-6. The results of the statistical analyses revealed that the addition of ASE did not significantly affect gas production from the soluble feed fractions (a), potentially degraded feed fractions (b), fractional rate of gas production (c), or total gas production (a + b) (p > 0.05). The ASE significantly affected methane production. Methane production was significantly reduced at an ASE level of 5% (p < 0.05).

**Table-6:** *In vitro* effects of ASE of *C. pubescens* on rumen gas production.

Parameter	Treatment group			

	P0 (ASE 0%)	P1 (ASE 1%)	P2 (ASE 2.5%)	P3 (ASE 5%)
Gas production from soluble feed fraction (a; mL/300 mg/dry matter)^ns^	10.83 ± 0.27	10.61 ± 0.19	10.34 ± 0.15	10.18 ± 0.14
Gas production from potentially degraded feed fractions (b; mL/300 mg/dry matter)^ns^	113.52 ± 0.34	113.27 ± 0.28	113.03 ± 0.13	112.92 ± 0.21
Fractional rate of gas production (c; mL/h)	0.05 ± 0.00	0.05 ± 0.00	0.05 ± 0.00	0.05 ± 0.00
Potential gas production (a + b; mL/300 mg dry matter)^ns^	124.35 ± 0.36	123.88 ± 0.27	123.37 ± 0.54	123.1 ± 0.24
CH_4_ (ppm)	13.72 ± 0.43^a^	11.94 ± 0.18^b^	10.03 ± 0.63^c^	8.31 ± 0.32^d^

ASE=Aqueous seed extract of *Carica* *pubescens*, P0=300 mg fermented substrate, P1=300 mg fermented substrate + 1% ASE, P2=300 mg fermented substrate + 2.5% ASE, P3=300 mg fermented substrate + 5% ASE. Data are presented as mean ± SD, ns=Not significant (p > 0.05). ^a,b,c^Different superscripts indicate significantly different means (p < 0.05)

## Discussion

Qualitative phytochemical analyses showed that the extract contained active compounds such as tannins, flavonoids, saponins, alkaloids, and steroids. Rungsung *et al*. [29] stated that plant parts are widely used as alternatives because they contain secondary metabolites and are believed to provide the maximum contribution to the treatment of several diseases. Shakya [30] reported that secondary plant metabolites include saponins, flavonoids, alkaloids, terpenoids, steroids, glucosides, tannins, and essential oils. Tungmunnithum *et al*. [31] asserted that plants commonly used as alternative medicines generally contain polar substances that are commonly referred to as plant bioactive components or secondary metabolites, including phenolic compounds and flavonoids.

Adama *et al*. [32] reported that *Anogeissus leiocarpus* and *Daniellia oliveri* plants containing tannins, saponins, and flavonoids exhibited an anthelmintic activity by suppressing adult *H. contortus*. Dubois *et al*. [33] reported that *Lupinus* spp. seed extract succeeded in inhibiting *H. contortus* due to active alkaloid compounds in the seeds. The death of these worms was most likely related to damage to various parts of the worm body (buccal area and cuticle) due to contact with metabolites in the ASE, as shown in Figure-1. Montellano *et al*. [34], Baihaqi *et al*. [35], and Baihaqi *et al*. [36] emphasized that the active compounds of tannin-rich plants cause damage to the body parts of the worm, including the cuticles, esophagus, reproductive tract, and buccal cavity. Barone *et al*. [37] reported that damage and aggregates were found in the buccal region of *H. contortus* that came into contact with CTs from cranberry vines. In contrast, worms that were not exposed to active compounds were observed to be normal. Tresia *et al*. [38] reported that anthelmintic activity is synergistically exerted by active compounds in plants by damaging the cuticle and changing the pore shape and permeability of the worm cuticle.

The results of this study are similar to those of Ampapon *et al*. [39], who stated that the addition of phytonutrient sources, such as mangosteen peel powder and banana flower powder containing CTs at 113–167 g/kg DW, did not significantly affect the pH, NH_3_, and VFA values *in vitro*. Similar findings were found *in vivo* where addition of 5% *Uncaria gambir* Roxb leaf residue containing 9.96% CTs did not alter the pH or total VFA content [27]. Suwignyo *et al*. [14] found that cattle fed the legume *Leucaena leucocephala* had no effect on pH, but increased propionate.

The pH of the rumen fluid resulting from the *in vitro* fermentation of feed in this study ranged from 6.81 to 6.86. The pH values were still in the normal range and were similar to the findings of Choudhury *et al*. [40], where the physiological pH value of the rumen fluid was 5.5–6.9. Meanwhile, the concentration of NH_3_ in the recent study ranged from 27.94 to 28.38 mg/100 mL and was within the physiological range (10.2–35.7 mg/100 mL) to support rumen microbial growth and metabolism as reported by McDonald *et al*. [41]. Furthermore, the average total VFA level was in the range of 101.91–104.32 Mm. McDonald *et al*. [41] stated that normal VFA levels of 70–150 Mm fulfill microbial cell protein synthesis requirements. The VFA concentration produced in this study generally met the rumen protein microbial synthesis requirements.

There was no significant effect of ASE treatment up to 5% on the microbial protein concentration. This could be related to the normal and unchanged NH3, VFA, and pH levels. The formation of microbial proteins requires a carbon skeleton derived from VFA and nitrogen from NH_3_ [42, 43]. Moreover, the addition of ASE did not significantly change the pH value of the rumen fluid (6.81–6.86; p > 0.05) and was, therefore, suitable for optimum microbial growth. The normal pH of rumen fluid is 5.5–7 [43,44]. Rumen pH above 5.7 is necessary for microbial protein synthesis, whereas pH above 6 allows for greater functional performance [45]. Kamra [46] stated that ruminal microbes grow optimally at pH 6–6.9. An *in vitro* study showed that the addition of tannin-containing 5% *U. gambir* extract in cattle feed supplement resulted in the optimum rumen pH (6.93–6.95) for microbial growth and protein synthesis [27]. In addition, Pathak [47] stated that tannin- and saponin-containing forage had greater efficiency in microbial protein synthesis.

The addition of ASE did not significantly affect gas production from the soluble feed fractions (a), potentially degraded feed fractions (b), fractional rate of gas production (c), or total gas production (a + b). These results are in accordance with those of previous studies reported by Yusuf *et al*. [28], which revealed that the addition of *Thevetia peruviana* containing active tannin compounds in the *in vitro* gas production test did not significantly affect fractions b and c. The rumen simulation technique described by Lins *et al*. [48] also showed that the addition of *Moringa oleifera* seeds containing tannin-active compounds had no significant effect on total gas production. Considering that gas production is an indicator of ruminal microbial fermentation or feed digestion rate [49], the results of the present study indicated that ASE does not interfere with microbial fermentation in the rumen.

The ASE significantly decreased methane production. This is in accordance with results reported by previous researchers. Niderkorn *et al*. [50] reported that CTs in *Carica avellana* with a concentration of 30.4% of the basal diet decreased methane production by 1.31 mmol/g DM. Similarly, studies on goats showed that feeding CT-containing *Sericea lespedeza* reduced methane emissions, which can be attributed to bacterial activity [51]. Moreover, a study on Bali cattle showed that supplementation with tannin-containing *U. gambir* Indonesia extract reduced methane production [52]. Studies in goats showed that applying saponin tea at a dose of 3 g/d reduced ruminal microbial fermentation and methane production but did not reduce the methanogen population [53]. Methanogen populations have decreased in dairy steers fed plants containing tannins and saponins [54]. The potency of numerous plant extracts for inhibiting rumen methanogens and CH_4_ production in ruminants has been previously reviewed by Baihaqi *et al*. [12], Patra *et al*. [55]. Demirtaş *et al*. [56] stated that the effects of plant secondary metabolites on ruminal fermentation are favorable if they increase or do not change VFA production (or with a desirable change in molar proportions of VFA) and feed digestibility while they decrease ammonia concentration and methane production. Ugbogu *et al*. [57] added that natural plant products or secondary metabolites have the potential to improve rumen fermentation, reduce feed energy loss, improve animal health and productivity, increase animal lifetime performance, and reduce GHG production (CH_4_ and CO_2_) during animal production. Furthermore, Halmemies-Beauchet-Filleau *et al*. [58] emphasized that ruminant-based food production faces multiple challenges, such as environmental emissions, climate change, and accelerating food–feed–fuel competition for arable land. Therefore, more sustainable feed production is required, along with the exploitation of novel resources. Cheng *et al*. [6] stated that climate is changing globally, affecting livestock. Climate affects livestock growth rate, milk and egg production, reproductive performance, morbidity, mortality, and feed supply. Simultaneously, livestock is a climate change driver, generating 14.5% of total anthropogenic GHG emissions. Based on these considerations, using up to 5% ASE containing secondary metabolites to suppress helminth infections and the production of GHGs provide a great advantage in improving health conditions, animal productivity, and environmental sustainability.

## Conclusion

Adding up to 5% ASE in the sheep diet demonstrated an anthelmintic activity against *H. contortus* and methane reduction without deleterious effects on rumen fermentation. This study provides a foundation for applying *C. pubescens* seeds in ruminant medicine and feeding. It also provides knowledge to improve animal health conditions and productivity and reduce GHG emissions. Therefore, further *in vivo* studies should be conducted to confirm the possible application of this medicinal plant in ruminants.

## Authors’ Contributions

ZAB, IW, WN, BS, and AAA: Designed the study and collected samples and performed laboratory examinations. ZAB: Conducted field surveys. All authors have drafted and revised the manuscript. All authors have read, reviewed, and approved the final manuscript.
